# Comparing Mental Strain and Subjective Sensations With and Without a Wearable Chair While Performing Simulated Suturing Tasks

**DOI:** 10.7759/cureus.69775

**Published:** 2024-09-20

**Authors:** Shoichi Shinohara, Kosuke Oiwa, Yoshitaka Maeda, Tsuneari Takahashi, Yuji Kaneda, Naohiro Sata, Hironori Yamaguchi, Hiroshi Kawahira

**Affiliations:** 1 Department of Surgery, Division of Gastroenterological, General and Transplant Surgery, Jichi Medical University, Shimotsuke, JPN; 2 Department of Information and Management Systems Engineering, Nagaoka University of Technology, Nagaoka, JPN; 3 Medical Simulation Center, Jichi Medical University, Shimotsuke, JPN; 4 Department of Orthopaedics, Jichi Medical University, Shimotsuke, JPN; 5 Medical Simulation Center/Department of Surgery, Division of Gastroenterological, General and Transplant Surgery, Jichi Medical University, Shimotsuke, JPN

**Keywords:** surgery, subjective sensation, heartrate variability, mental strain, exoskeleton, wearable chair

## Abstract

Introduction

Prolonged standing during surgery is a cause of musculoskeletal disorders in surgeons. Wearable chairs have reportedly reduced musculoskeletal strain effectively when used in industry. However, discomfort and instability issues may remain. This study examines whether using a wearable chair for surgical procedures imposes negative effects, such as mental or physical strain on surgeons prior to its clinical implementation.

Methods

This prospective cross-over study compared mental strain and subjective sensations in simulated suturing tasks with and without using Archelis®, a wearable chair (Archelis Inc, Yokohama, Japan). Six surgeons participated in the study. Mental strain was examined using heart rate variations calculated during tasks. Four subjective sensations (fatigue, comfort, balance, and workability) after each task and differences in body localized pain pre- and post-task were compared using a 10 centimetres (cm)-visual analog scale (VAS) score questionnaire.

Results

Results showed no significant differences in mental strain nor subjective sensations with or without the wearable chair. The mean VAS scores for all four subjective sensations with the wearable chair were relatively positive. There was a slight yet insignificant post-task increase in VAS mean scores for body-localized pain in the lower legs.

Conclusions

Significant negative effects on surgeons from the wearable chair were not observed during simulated suturing procedures. This demonstrates no major barriers in the initial phases of wearable chairs integration into the surgery environment.

## Introduction

Surgeons are required to stand for long periods of time during surgery. Prolonged standing is known to have adverse health effects, such as musculoskeletal disorders, and surgeons have complained of neck, shoulder, and lower back pain [1-4]. To improve this operating environment, some surgical procedures are performed while sitting in a chair [5]. However, if moving or adjusting the chair during operations, there are risks of falls or other need for assistance.

Wearable chairs (or passive exoskeletons) may present one solution. In industry, wearable chairs reduce the accumulation of lower limb fatigue and are effective in reducing strain for longer periods of time [6-8]. Wearable chairs are also suitable in environments with limited space around other objects because they are compact and follow the user’s body. However, some studies have reported negative effects of wearable chairs, including discomfort, increased unexpected muscle activity, and risk of falls [7,9,10].

When introducing a new technology, it is necessary to ensure that any potential risks such as discomfort are not greater than those associated with conventional methods. While there has been a study evaluating the influence of surgical procedures using wearable chairs on scopists, to the best of our knowledge there are no studies assessing the impact on surgeons [11]. Therefore, we sought to determine how the use of a wearable chair for surgical procedures might affect mental strain and subjective sensations related to usability before introducing for clinical use. As an initial evaluation, this study focused on short and simple simulated suturing procedures in a laboratory environment.

## Materials and methods

Study design

This prospective cross-over study comparing mental strain and subjective sensations in simulated suturing tasks with and without a wearable chair was conducted between November 2020 and March 2021 at the Medical Simulation Center, where the co-authors were affiliated. This study was approved by the Institutional Ethics Committee of Jichi Medical University on March 29, 2019 (Approval number: A18-158).

A wearable chair

**Figure 1:**
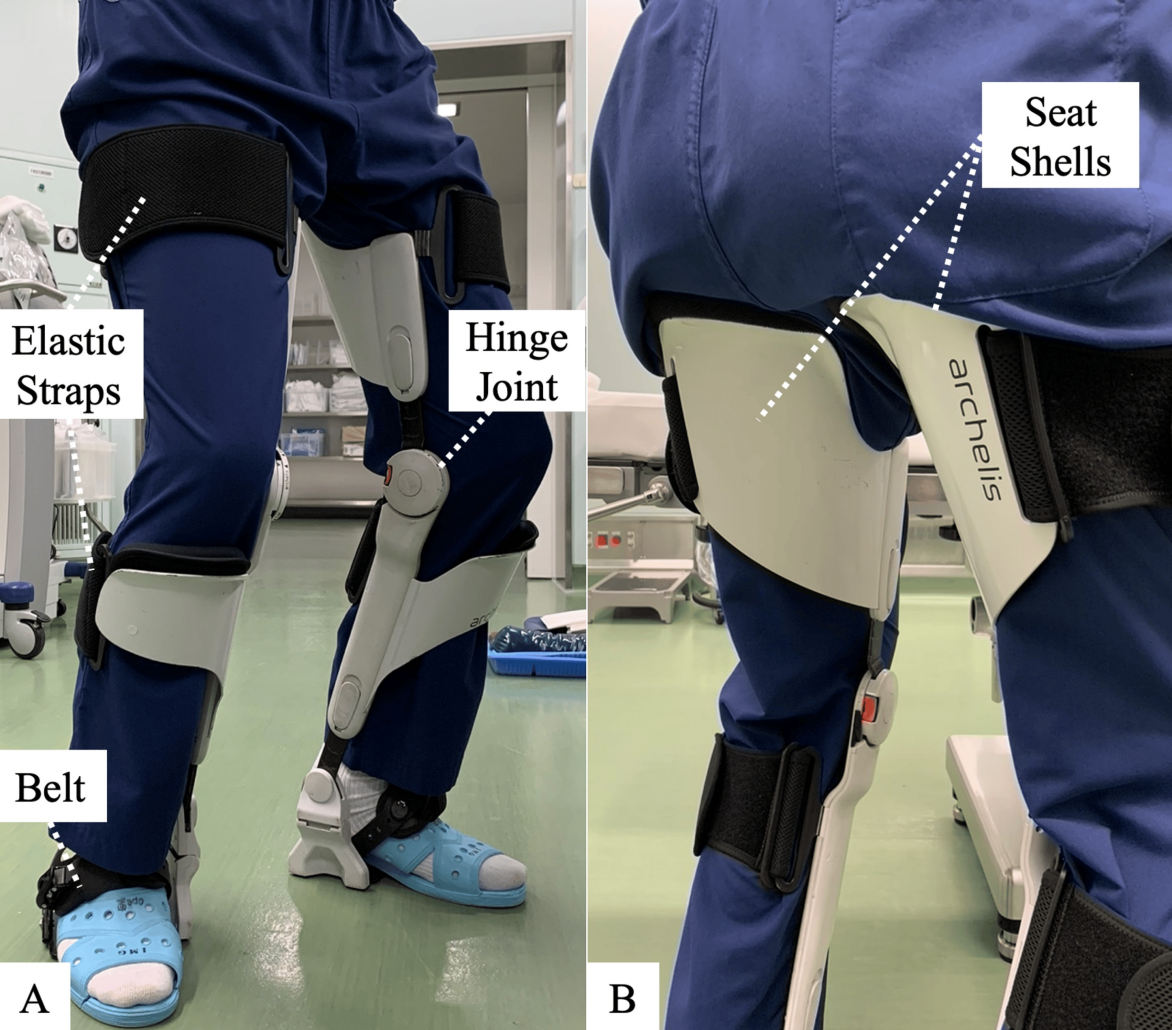
Archelis®, a wearable chair Archelis® (Archelis Inc, Yokohama, Japan) was used in this study. A participant is wearing Archelis® on their lower extremities: (A) the front view and (B) the back view.

For this study, the Archelis® wearable chair (Archelis Inc, Yokohama, Japan), previously evaluated favorably among scopists was used (Figure 1) [11]. The chair consists of two metal supports attached to the inside of the user’s legs. Each support can be adjusted in length with eight different settings and has a hinge joint at knee level and seat shells below the buttocks. The supports are secured to the user’s feet using belts, and they are also attached to the lower legs and thigh with elastic straps. It allows the user to sit in a high sitting position, and part of the physical load (i.e., body weight) carried by the lower limbs can be transferred to the ground by the supports. It does not require electricity or power. The total weight of each side is approximately 2.7 kg with a maximum load capacity of 90 kg.

Participants

Surgeons who fulfilled weight and height requirements (weight≦90 kg, height≦185 cm) and were experienced with abdominal surgery were recruited. Prior experience with the chair was not required. Exclusion criteria included a history of cardiac disease and arrhythmia, musculoskeletal and systemic disorders, and any known impairments in postural control or motor function.

Organization of the procedure

**Figure 2:**
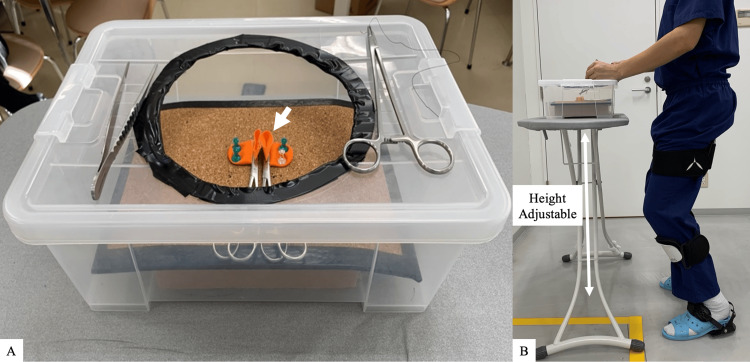
The intestinal anastomosis model (A) This model used finger cots (white arrow) (B) Each participant chose the most appropriate height of table.

Before the experiment, signed informed consent statements were obtained after each participant was informed of the purpose and procedure and how to wear the chair. Suturing tasks were model-simulated intestinal anastomosis mimicking a surgical environment (Figure 2). All participants used pre-work activities for 10 minutes to become familiar with the wearable chair and experimental task. Participants freely adjusted the working distance or table height for each task. Participants performed a series of six stitches on a simulated intestinal tract with and without the wearable chair in sequence assigned by the researcher. The duration of each task from start to finish of stitching was measured on a digital timer. A 10-minute break was taken between tasks [12]. The order of the two conditions was allocated to the participants by the researcher design for the equal number of conditions.

Analysis performed

Mental Strain Assessment

Mental strain was evaluated by heart rate variability (HRV), the fluctuation of the length of heartbeat intervals (RR intervals) [13,14]. RR intervals were recorded during each task with an eMotion Faros 90° heart rate recorder (Mega Electronics Ltd, Kuopio, Finland). The power spectral density was calculated with fast Fourier transformation of the data resampled from the recorded RR interval data at a sampling frequency of 20 Hz [15]. High-frequency (HF, 0.15-0.4 Hz) and a low-frequency (LF, 0.04-0.15 Hz) power spectral density were calculated from the data. HF increases in response to vagal activity and LF increases in response to sympathetic activity [16]. LF/HF ratio is a measure of HRV and represents an indirect measure of mental strain. An increase in the ratio represents increase strong in mental strain through strong arousal and tension with little cardiac relaxation [17].

Subjective Sensations Assessment

The 10 centimetres (cm)-visual analog scale (VAS) was marked by participants after each task to evaluate subjective fatigue, comfort, balance, and workability [18]. Difference in scores before and after each task measured subjective body-localized pain in eight anatomical regions: neck, shoulder, arm, lower back, hips, thigh, lower leg, foot [19]. For fatigue, comfort, balance, and workability, the score ranged from -5 on the left border to 5 on the right border of the scale: “-5”represented the severest levels of fatigue, discomfort, instability, and task difficulty; and “5” represented highest levels of vigor, comfort, stability, and task facility. The midpoint at 0 represented a neutral state. For subjective body-localized pain, the score ranged from 0 at the left to 10 at the right, with “0” representing no pain and “10” representing the worst possible pain.

Statistical analyses

Priori power analysis was performed using G* Power 3.1 (Franz Paul, Kiel, Germany) [20]. The sample size was calculated with 80% power and an effect size of 4.9 to test the study hypothesis [21]. Based on this calculation, we confirmed that the sample size of the study (n = 12) was sufficient to determine the statistical significance. All statistical analyses were performed using R statistical software (version 4.3.0). For continuous data, mean ± standard deviation or median (interquartile range) were used as applicable. Mann-Whitney U test was performed to evaluate the difference between the groups. The level of significance was set at P < 0.05.

## Results

Six surgeons participated (females = one, total mean body height 173.3 ± 5.7 cm, weight 71.2 ± 5.8 kg, Table 1) in this study. In total, 12 tasks (six tasks with and six tasks without the chair) were analyzed. Four surgeons held board certification in Japan, and the other two were in surgical residency training. Two surgeons had previous experience using the wearable chair during surgery.

**Table 1:** Surgeon’s characteristics

No.	Gender	Body Height, cm	Body Weight, kg	Board Certification	Previous Experience with Wearable Chair
1	Male	176	81	Yes	No
2	Male	165	67	Yes	Yes
3	Male	176	65	Yes	Yes
4	Female	166	70	No	No
5	Male	177	67	No	No
6	Male	180	77	Yes	No

To check whether rest values returned to baseline before starting a new task, heart rate was measured within one minute before each task. The mean heart rate before the initial task was 80.5 ± 9.0 beats per minute (bpm) and it was 80.0 ± 8.6 bpm per minute before the subsequent task (no statistical difference, Z = 0.39, p = 1.00). The mean duration of the tasks was around 4 minutes with no significant difference between the groups (245 ± 60 sec with the wearable chair 228 ± 58 sec without the wearable chair; Z = 0.64, p = 0.56).

LF/HF ratio during the task

**Figure 3:**
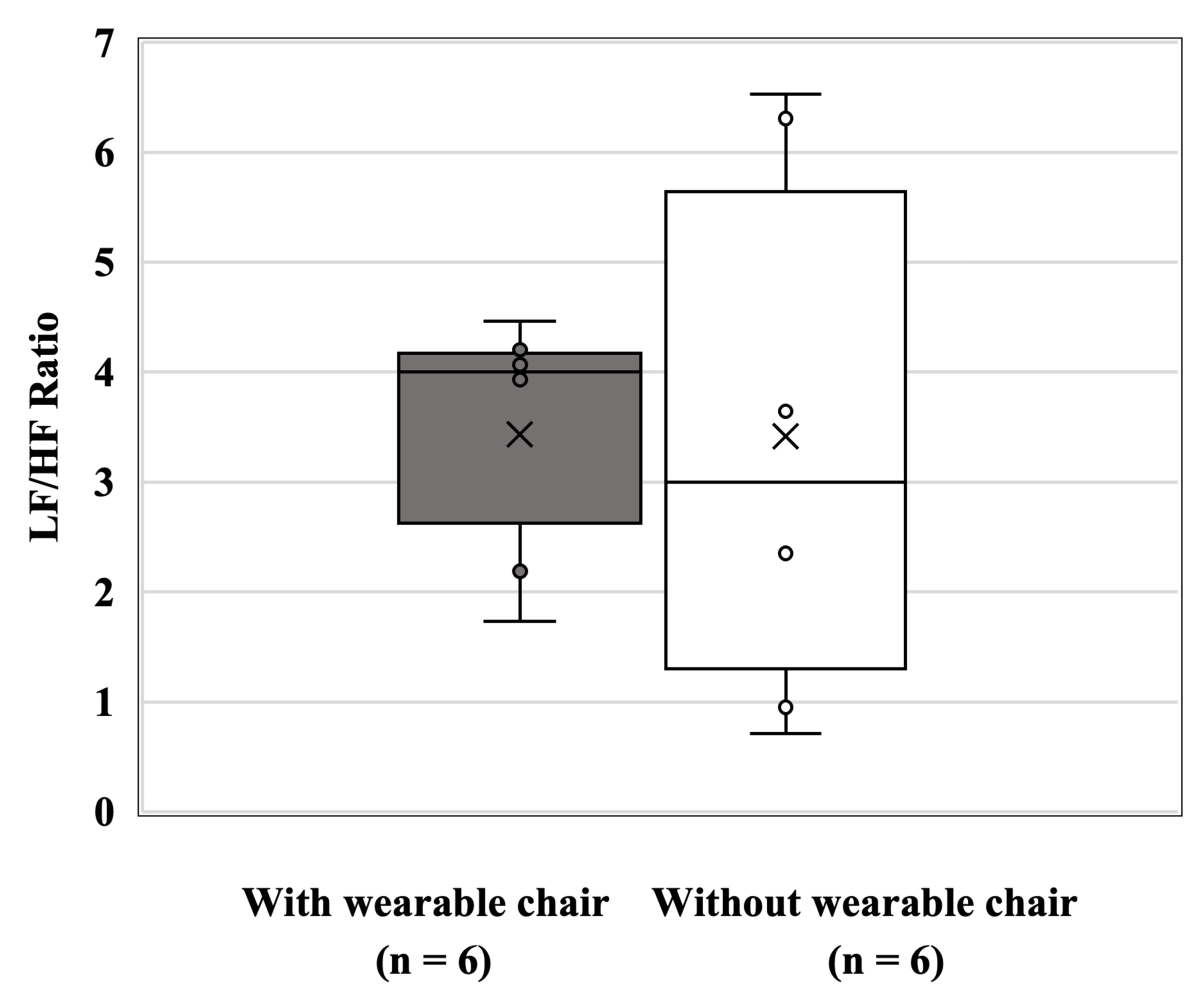
Box and whisker plot of LF/HF ratio comparison between postures with and without Archelis. The box and whisker plot show median values, interquartile range, and spread for the ratio of LF /HF in relation to the two postures (with/without the wearable chair). “×“ indicates the mean of each group. “○“ marks the value of the individuals.

The LF/HF ratio during the task was 3.43 ± 1.06 with the wearable chair and 3.41 ± 2.33 without the wearable chair. No significant difference was observed between the groups (Z = 0.32, p = 0.82) (Figure 3).

Difference in body-localized pain on the 100 mm-VAS scores

**Figure 4:**
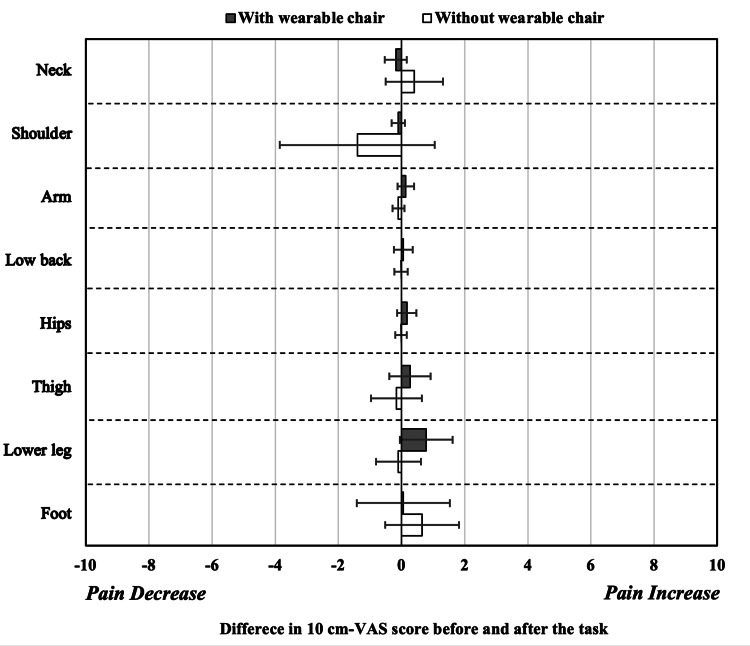
Mean difference in body-localized pain on 10 cm-VAS scores comparison between postures with and without the wearable chair. The mean difference in body-localized pain on the 10 centimetres (cm)-visual analog scale (VAS) scores before and after the task with the wearable chair (n = 6, error bar: standard deviation (SD) and the task without the wearable chair (n = 6, error bar: SD). Black bars: with the wearable chair; white bars: without the wearable chair. A score of 0 indicates no change in the VAS score before and after the task. A score of 10 at the right edge indicates a 10 increase in body-localized pain on the VAS score after the task, while a score of -10 at the left edge indicates a 10 reduction in pain after the task. The mean of the differences in values before and after the task is shown.

There was no difference for all difference in the VAS scores for body-localized pain (Figure 4). The increase in the VAS score after the wearable chair use task was most pronounced in the lower legs, but the mean increase was only 0.8. However, there were variations for the thigh, lower leg, and foot.

100 mm-VAS scores for fatigue, comfort, balance, and workability

**Figure 5:**
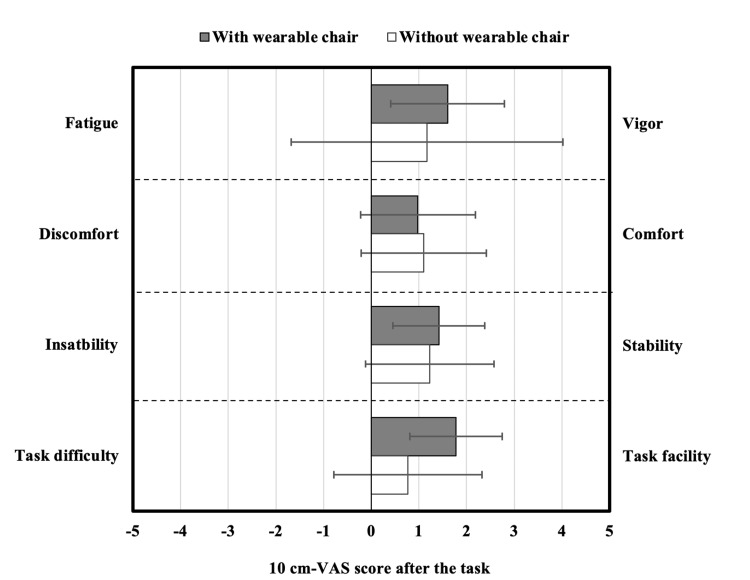
Mean 10 cm-VAS scores comparison between postures with and without the wearable chair. Mean 110 centimetres (cm)-visual analog scale (VAS) scores after the task with the wearable chair (n = 6, error bar: SD) and the task without the wearable chair (n = 6, error bar: SD). Black bars: with the wearable chair; white bars: without the wearable chair. The score at the left-hand end of the VAS is defined as -5, and the score at the right-hand end as 5. A score of 0 in the middle means neither. The mean value after the task is shown.

There was no significant difference in the VAS scores for fatigue (Z = -0.32, p = 0.82), comfort (Z = 0.08, p = 0.97), balance (Z = -0.32, p = 0.82), and workability (Z = 1.52, p = 0.15) (Figure 5). The mean VAS scores for each of the four subjective sensations with the wearable chair were not less than 0.

## Discussion

The most important findings of the current study were that the mental strain on the surgeon was not significantly affected using the wearable chair. Notably, subjective body localized pain was only slightly aggravated and there was little negative effect on the subjective fatigue and comfort. Additionally, there were no negative effects on the subjective balance and workability. In other words, there did not appear to be major barriers to surgeon acceptance during this introductory session.

In past reports, wearable devices caused discomfort and pain if the device did not fit the human body or interfered with movement [9,22,23]. In other studies, users of wearable chairs experienced discomfort when the working distance or height was inappropriate [24]. Findings here suggested no negative impacts on suturing procedures in an environment where height and distance can be freely adjusted, such as a surgical setting. This could explain non-significant differences in the mental strain such as the LF/HF ratio. In addition, the minimal exacerbation of pain despite individual variations in body-localized pain might be a factor that did not lead to significant deterioration in subjective comfort.

There were non-significant negative effects on the subjective stability suggesting the wearable chair was acceptable if limited to suturing procedures. Motion restriction and inability to maintain posture have been reported to cause feelings of instability, although the use for surgical assistants, scrub nurses and rehabilitation patients showed favorable stability [7,9,11,19,25,26].

There were also no outstanding negative effects on subjective workability. User posture, with slightly flexed knees that closely resembled a standing posture, was normal for surgery. This may explain the results here for regular suturing. In a previous study, exoskeletons had positive effects on productivity [27].

Our study has several limitations. First, as an experimental study, the results cannot be applied to all clinical settings. Mental strain and subjective sensations may differ between clinical and experimental conditions. Second, this study was conducted with short-term suturing procedures. Therefore, the use of the wearable chair for long periods of time and surgical techniques other than suturing were not examined. Third, the sample size was small and should be increased in later studies.

## Conclusions

The study suggested no significant differences in mental strain and subjective sensations for surgeons with or without the wearable chair, and during simulated surgical procedures with the chair, no notable negative effects were observed. These results suggested wearable chairs may be potentially acceptable as devices enabling surgeons to perform surgical procedures with no diminution in sensation compared to standing postures. This shows the potential for clinical use of the wearable chair by surgeons.
